# Persistent postural–perceptual dizziness: subjective–objective dissociation and response to neurologist-led multimodal therapy

**DOI:** 10.1007/s00415-026-13927-6

**Published:** 2026-06-10

**Authors:** Sudhir Kothari, Darshan Bhansali, Deepak S. Phalgune, Nilesh Bhandari, Ajay Kumar Vats, Diego Kaski

**Affiliations:** 1 Poona Hospital & Research Center, Pune, Maharashtra India; 2 Prakriti Hospital Virar West, Virar, Maharashtra India; 3 Shantiraj Hospital, Udaipur, Rajasthan India; 4 UCL Institute of Neurology, National Hospital for Neurology and Neurosurgery, London, UK

**Keywords:** Persistent postural–perceptual dizziness, Functional neurological disorder, Vestibular rehabilitation, Cognitive behavioral therapy, Anxiety, Posturography

## Abstract

**Background and objectives:**

Persistent postural–perceptual dizziness (PPPD) is characterized by maladaptive central sensory processing and frequent psychiatric comorbidity. Prospective outcome data, particularly from low- and middle-income settings, remain limited. This study evaluated clinical characteristics, psychiatric comorbidity, objective balance findings, and treatment response in patients with PPPD, with emphasis on neurologist-delivered diagnostic explanation within a multimodal treatment framework.

**Methods:**

Seventy-five consecutive patients fulfilling Bárány Society criteria for PPPD were prospectively assessed using the Dizziness Handicap Inventory (DHI), Patient Health Questionnaire-9 (PHQ-9), Generalized Anxiety Disorder-7 (GAD-7), and Balance Rehabilitation Unit posturography. All patients received structured, neurologist-delivered diagnostic explanation and education. Treatment consisted of vestibular rehabilitation and cognitive behavioral therapy for all patients, with low-dose antidepressant therapy prescribed when PHQ-9 or GAD-7 scores were ≥ 5. Outcomes were reassessed at 3 months.

**Results:**

Psychiatric comorbidity was present in 88% of patients, most commonly combined anxiety and depression. Despite moderate to severe subjective disability, objective balance measures were largely normal, demonstrating a characteristic subjective–objective dissociation. Following treatment, median DHI scores improved from 40.4 to 8.0, PHQ-9 from 8.0 to 0.0, and GAD-7 from 8.0 to 0.0 (all *p* < 0.001).

**Conclusions:**

PPPD is associated with high psychiatric comorbidity and marked subjective–objective dissociation yet shows substantial 3-month clinical improvement with a structured multimodal treatment approach. Structured diagnostic explanation and education may facilitate treatment engagement and recovery.

## Introduction

Persistent postural–perceptual dizziness (PPPD) is a common cause of chronic dizziness characterized by maladaptive central processing of vestibular, visual, and somatosensory information. It reflects dysfunction within distributed sensory and limbic networks rather than ongoing peripheral vestibular pathology. The condition was formally defined by the Bárány Society in 2017, consolidating earlier entities such as phobic postural vertigo, space–motion discomfort, and chronic subjective dizziness into a single diagnostic construct [1, 2]. This consensus marked a shift toward understanding PPPD as a disorder of central sensory integration, now classified within the spectrum of functional neurological disorders.

The Bárány Society criteria define PPPD as persistent non-spinning dizziness or unsteadiness present on most days for at least three months, exacerbated by upright posture, motion, or complex visual environments, and often—but not invariably—precipitated by an acute vestibular, medical, or psychological event [2]. PPPD is a positive diagnosis based on characteristic clinical features rather than one of exclusion, and idiopathic onset is explicitly recognized.

Current pathophysiological models emphasize maladaptive sensory reweighting, with excessive reliance on visual and somatosensory inputs and reduced integration of vestibular signals [3]. Heightened threat appraisal, anxiety, and hypervigilance further amplify this imbalance, creating a self-perpetuating cycle. Neuroimaging studies support this network-based model, demonstrating altered activity and connectivity within vestibular–visual–limbic circuits, including the insula and temporoparietal junction [4–7]. These findings reinforce PPPD as a disorder of central sensory network regulation rather than peripheral vestibular dysfunction [8, 9].

Psychiatric comorbidity, particularly anxiety and depression, is commonly reported in PPPD and is thought to influence symptom persistence and severity [10, 11]. Contemporary models conceptualize affective symptoms as modulators of sensory processing and threat appraisal rather than primary causal drivers [1, 9].

PPPD is increasingly recognized as a frequent cause of chronic dizziness in specialized clinics, accounting for a substantial proportion of presentations in published series [1, 9]. It is associated with significant impairment in quality of life, often exceeding that seen in other chronic vestibular disorders [11]. Although a female predominance is well described, substantial prevalence in men has also been reported [2, 12]. Despite its frequency, PPPD remains under-recognized in routine neurological and otolaryngological practice, particularly in low- and middle-income settings, where prospective outcome data remain limited [1, 13]. In addition, the evidence base for PPPD treatment remains relatively underdeveloped. Recent Cochrane reviews highlighted the limited number of controlled randomized studies and the heterogeneity of both pharmacological and non-pharmacological treatment protocols [14, 15]. Most available evidence derives from retrospective or small prospective cohorts, underscoring the need for systematic prospective outcome studies [16].

Increasing attention has been directed toward the role of diagnostic explanation and patient education in PPPD management. Clear communication of the diagnosis, provision of a coherent explanatory model, and expectation-setting are now regarded as foundational elements of care[1, 9]. This approach aligns with broader functional neurological disorder literature, which recognizes the neurological consultation itself—when it provides a clear, positive diagnosis and mechanistic explanation—as an early therapeutic intervention rather than a mere diagnostic step. Such structured education facilitates acceptance, reduces maladaptive threat responses, and enhances engagement with rehabilitation and psychological therapies [17, 18].

Although prior studies, including Waterston et al., have demonstrated improvement in PPPD using cognitive–behavioral therapy and multimodal approaches, treatment protocols remain heterogeneous and the contribution of physician-delivered diagnostic explanation has not been systematically examined [16]. Furthermore, prospective outcome data from low- and middle-income clinical settings remain limited. These gaps highlight the need for structured prospective studies evaluating integrated treatment approaches in routine neurological practice.

On this basis, we implemented a structured, neurologist-led multimodal treatment approach for PPPD, integrating diagnostic explanation, vestibular rehabilitation, targeted psychological intervention, and pharmacotherapy when indicated. We hypothesized that clear neurological framing of the diagnosis, combined with integrated therapy, would improve adherence and clinical outcomes.

The present study prospectively evaluated the clinical characteristics, psychiatric comorbidities, objective balance findings, and treatment response in patients with PPPD. By examining subjective–objective dissociation and outcomes following a neurologist-led multimodal intervention, this study aims to provide clinically relevant data to inform PPPD management in routine neurological practice.

## Methods

### Study design and participants

This prospective observational study was conducted at a tertiary neurology clinic specializing in dizziness and balance disorders between September 2021 and June 2023. Consecutive patients aged ≥ 18 years who fulfilled the Bárány Society diagnostic criteria for PPPD were enrolled at the time of first diagnosis after written informed consent.

The diagnosis of PPPD was made by the treating neurologist, who has specialist expertise in dizziness and balance disorders, using the Bárány Society criteria. The diagnosis was based on characteristic positive clinical features rather than exclusion alone.

Patients with active peripheral vestibular disorders causing ongoing vestibular symptoms, structural or degenerative neurological disorders capable of independently explaining chronic dizziness or imbalance—including cerebellar disorders, neurodegenerative disease, or active disabling stroke-related deficits—or severe psychiatric illness requiring inpatient care were excluded. Migraine, including vestibular migraine, was not an exclusion criterion, as it is a recognized precipitating or comorbid condition in PPPD.

All patients underwent detailed neurological and neuro-otological clinical evaluation. Vestibular investigations, including vestibular function testing when clinically indicated, were performed to exclude active vestibular pathology or identify associated vestibular disorders.

### Clinical and psychiatric assessment

All participants underwent detailed clinical evaluation, including structured history-taking and neurological examination, with emphasis on symptom characteristics, duration, precipitants, and exacerbating factors.

Psychiatric symptoms were screened using the Patient Health Questionnaire-9 (PHQ-9) for depressive symptoms and the Generalized Anxiety Disorder-7 (GAD-7) scale, using validated cut-offs to classify severity [19, 20]. The impact of dizziness on daily functioning was assessed using the Dizziness Handicap Inventory (DHI), a 25-item patient-reported outcome measure with total scores ranging from 0 to 100, categorized as mild (0–30), moderate (31–60), or severe (61–100) [21].

### Posturography

Postural stability was evaluated using the Balance Rehabilitation Unit (BRU™, Medicaa) platform under six standardized sensory conditions [22]. All BRU-derived parameters were examined for association with DHI scores; however, only the Limit of Stability (LOS) demonstrated sufficient consistency for meaningful subgroup analysis.

### Diagnostic explanation and education

All patients received structured, neurologist-delivered diagnostic explanation and education in accordance with published recommendations. PPPD was explicitly presented as a positive, brain-based diagnosis based on established criteria, rather than a diagnosis of exclusion. The explanation emphasized maladaptive central sensory processing following a resolved precipitating event, when present, and the role of hypervigilance and altered balance strategies in symptom persistence.

The rationale for treatment components was explained in clear, mechanistic terms: vestibular rehabilitation therapy (VRT) to retrain sensory integration and balance strategies; psychological intervention to reduce maladaptive threat appraisal and avoidance behaviors; and pharmacotherapy, when indicated, to reduce anxiety or depressive symptoms and facilitate recovery. Explanations were tailored to individual clinical histories, and supplementary written material was provided when appropriate.

### Multimodal therapy

All patients were prescribed individualized VRT supervised by trained physiotherapists, including gaze stabilization, habituation, and balance exercises. Cognitive-behavioral therapy (CBT) was offered in 2–4 sessions by a licensed clinical psychologist, focusing on psychoeducation, cognitive restructuring, and graded exposure to symptom-provoking situations.

Pharmacotherapy was initiated only in patients with clinically significant psychiatric symptoms, defined as PHQ-9 or GAD-7 scores ≥ 5.

Patients already receiving antidepressants, anxiolytics, or vestibular suppressants at presentation were not excluded solely on that basis, provided they fulfilled Bárány Society criteria for PPPD and did not have severe psychiatric illness requiring inpatient care. Medication history was recorded, and treatment was reviewed and rationalized as part of the multimodal protocol.

Low-dose antidepressant therapy was prescribed when clinically indicated. Medications included escitalopram 5–10 mg, paroxetine 12.5 mg, or mirtazapine 7.5–15 mg, with careful titration and monthly monitoring for adherence and tolerability.

### Outcome assessment

Clinical reassessment and repeat DHI, PHQ-9, and GAD-7 assessments were performed at 3 months. Adherence to therapy and adverse events were recorded at follow-up visits.

### Ethics

The study was approved by the Institutional Ethics Committee (RECH/ECBHR/2020–21/262; 27 August 2021) and conducted in accordance with the Declaration of Helsinki. Written informed consent was obtained from all participants.

### Statistical analysis

Categorical variables are presented as numbers and percentages. Continuous variables are presented as the mean and standard deviation (SD), and median with interquartile range (IQR) for parametric and non-parametric data, respectively. As outcome measures were non-normally distributed, pre- and post-treatment comparisons for DHI, PHQ-9, and GAD-7 were performed using the Wilcoxon signed-rank test. Fisher’s exact test was used for categorical comparisons. Statistical analyses were performed using SPSS version 24.0 (IBM Corp., Armonk, NY, USA), and a p-value < 0.05 was considered statistically significant.

## Results

### Patient characteristics

Seventy-five patients met the diagnostic criteria for PPPD and were included in the study. The mean age was 46.8 ± 13.2 years, with a female predominance (70.7%). Most patients (88%) had symptom duration ≤ 3 years. Demographic and clinical features, including age distribution, gender, symptom duration, and baseline severity of dizziness and psychiatric symptoms, are summarized in Table 1.

**Table 1:** Demographic and clinical features, including age distribution, gender, symptom duration, and baseline severity of dizziness and psychiatric symptoms

Variables	Category	*n* (%)
Age in years (Mean ± SD)		46.8 ± 13.2
Age distribution in years	≤ 30	12 (16.0)
	31–40	13 (17.3)
	41–50	19 (25.3)
	51–60	18 (24.0)
	61–70	13 (17.4)
Gender	Male	22 (29.3)
	Female	53 (70.7)
Duration of symptoms	< 1 year	33 (44.0)
	1–3 years	33 (44.0)
	> 3 years	9 (12.0)
Baseline DHI severity	Mild (0–30)	23 (30.7)
	Moderate (31–60)	37 (49.3)
	Severe (61–100)	15 (20.0)
PHQ-9 severity	None–minimal (0–4)	12 (16.0)
	Mild (5–9)	30 (40.0)
	Moderate (10–14)	27 (36.0)
	Severe (≥ 15)	6 (8.0)
GAD-7 severity	None–minimal (0–4)	23 (30.7)
	Mild (5–9)	27 (36.0)
	Moderate (10–14)	17 (22.7)
	Severe (≥ 15)	8 (10.7)

*DHI* Dizziness Handicap Inventory, *PHQ* Patient Health Questionnaire, *GAD* Generalized Anxiety Disorder

### Precipitating events

A precipitating event was identified in 43 patients (57.3%). The most common precipitating vestibular disorder was BPPV (36/43, 83.7%), followed by syncope (*n* = 3, 7.0%), trauma (*n* = 2, 4.7%), vestibulopathy (*n* = 2, 4.7%), and stroke (*n* = 1, 2.3%). One patient (2.3%) had another precipitant, while the remaining 32 patients (42.7%) had idiopathic onset. These findings are presented in Table 2.

**Table 2:** Precipitating events in patients with PPPD (*n* = 75)

Precipitating Event	Number of cases	% of Triggered Cases (*n* = 43)	% of Total Cohort (*n* = 75)
Patients without precipitating event			
Idiopathic onset	32	NA	42.7%
Patients with identifiable precipitating event			
BPPV	36	83.7%	48.0%
Syncope	3	7.0%	4.0%
Trauma	2	4.7%	2.7%
Vestibulopathy	2	4.7%	2.7%
Stroke	1	2.3%	1.3%
Other	1	2.3%	1.3%
Subtotal (Triggered)	43	100%	57.3%

*BPPV* benign paroxysmal positional vertigo

### Baseline handicap and psychiatric comorbidity

At baseline, the median DHI score was 40.4 (range: 18–74), corresponding to moderate disability. By severity category, 30.7% of patients had mild, 49.3% moderate, and 20.0% severe handicap.

**Fig. 1:**
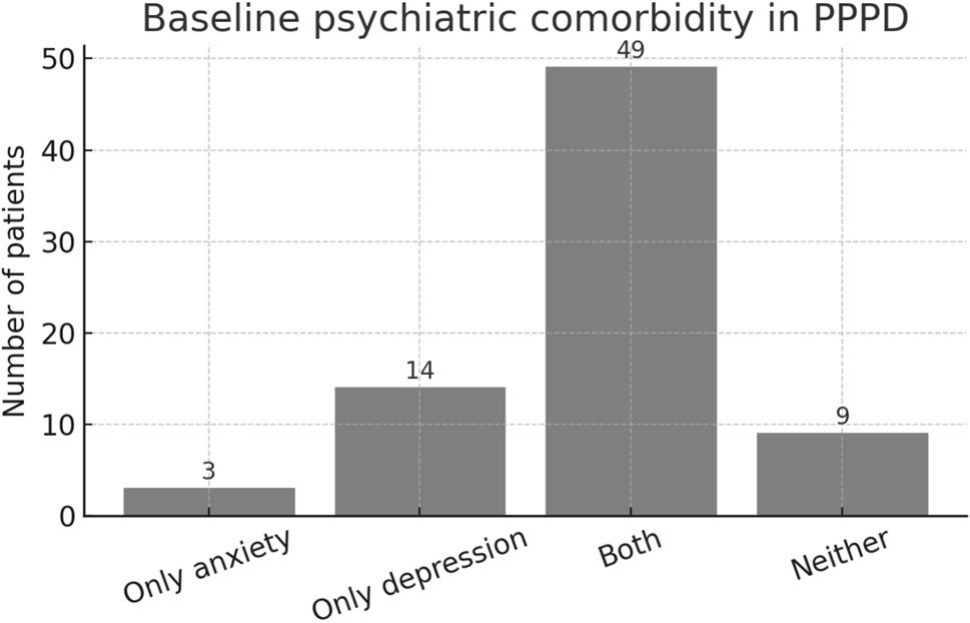
Distribution of psychiatric comorbidity in patients with PPPD, categorized as combined anxiety and depression, anxiety only, depression only, or neither

Psychiatric screening revealed that 49 patients (65.3%) had both anxiety and depression, 3 (4.0%) had anxiety only, 14 (18.7%) had depression only, and 9 (12.0%) had neither. The distribution of psychiatric comorbidity is illustrated in Fig. 1.

Patients with identifiable precipitating events had significantly higher baseline psychiatric symptom scores than those with idiopathic onset (median PHQ-9: 11.5 vs. 8.0, *p* = 0.021; median GAD-7: 10.0 vs. 6.0, *p* = 0.049), while baseline DHI scores did not differ between the two groups (*p* = 0.271).

### Subjective–objective mismatch

**Fig. 2:**
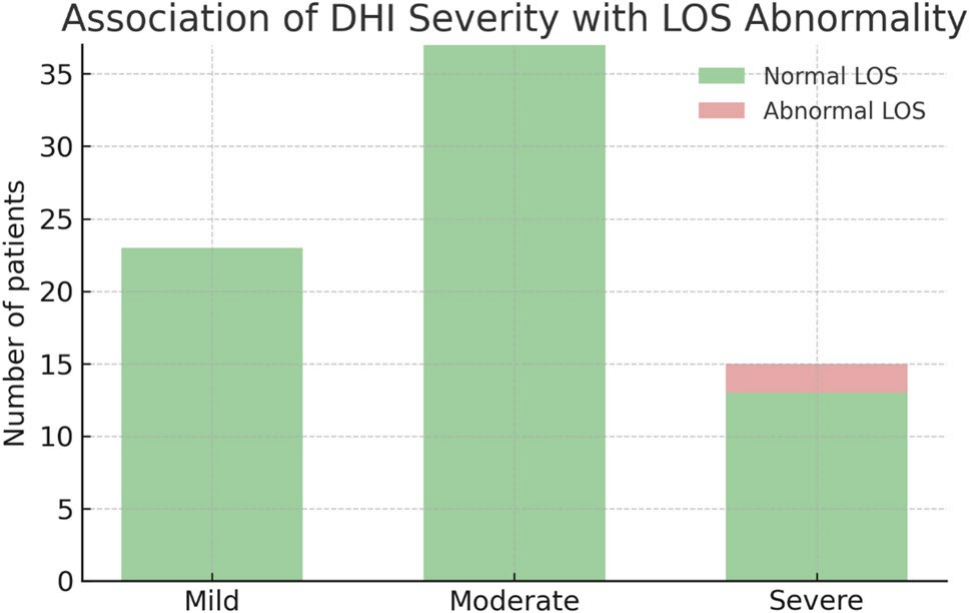
Association between DHI severity categories (mild, moderate, severe, x-axis) and Limit of Stability (LOS) status in patients with PPPD. Bars represent the number of patients with normal and abnormal LOS within each DHI severity category

Despite substantial subjective disability, objective balance performance was largely preserved. BRU™ testing showed that most posturographic parameters—including sway velocity, ellipse area, and sensory condition-specific stability indices—were within normal limits at the group level and showed no significant correlation with baseline DHI scores. Limit of stability (LOS) was the only parameter to demonstrate abnormal values, observed in 2 of 75 patients (2.7%). All patients with mild or moderate DHI scores had normal LOS, whereas 2 of 15 patients (13.3%) with severe DHI exhibited abnormal LOS. The association between DHI severity and LOS abnormality was statistically significant (*p* = 0.016; Fig. 2). These findings underscore the characteristic dissociation in PPPD between marked self-reported disability and largely preserved objective balance function.

### Treatment outcomes

**Fig. 3:**
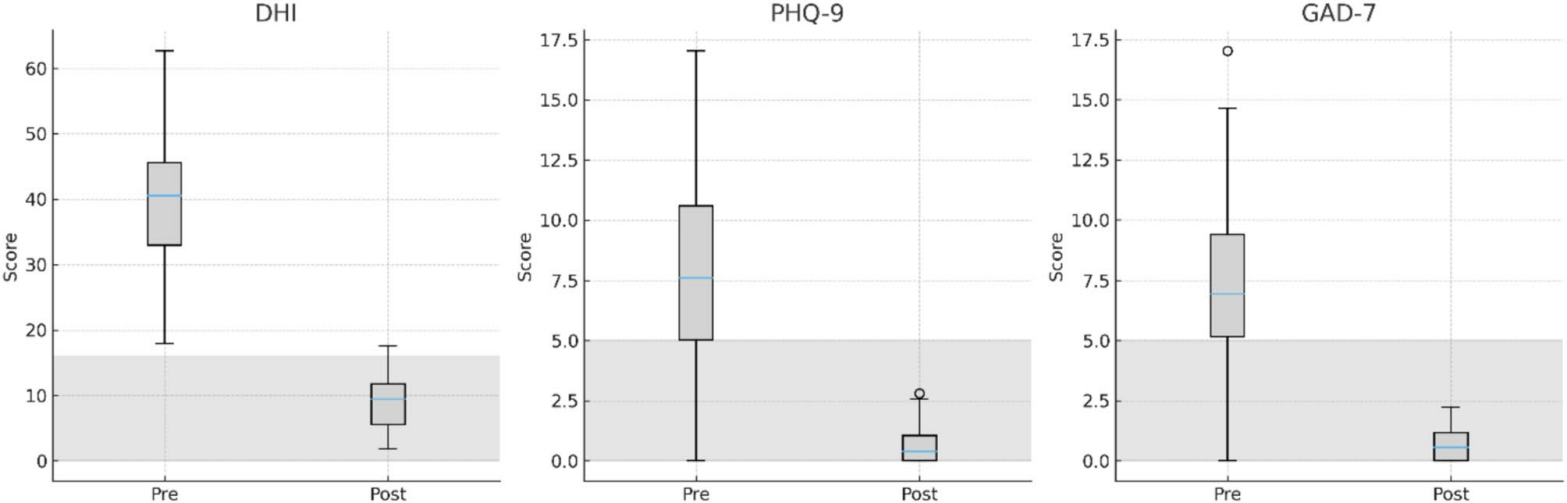
Pre- and post-treatment DHI, PHQ-9, and GAD-7 scores after 3 months of structured counselling and multimodal therapy. Boxplots show medians, interquartile ranges, and full ranges

After 3 months of structured counselling and multimodal therapy, significant improvement was observed across all outcome measures. Median DHI scores decreased from 40.4 to 8.0, PHQ-9 scores from 8.0 to 0.0, and GAD-7 scores from 8.0 to 0.0 (all p < 0.001). Interquartile ranges demonstrated that the majority of patients shifted into the minimal symptom range (Fig. 3).

**Fig. 4:**
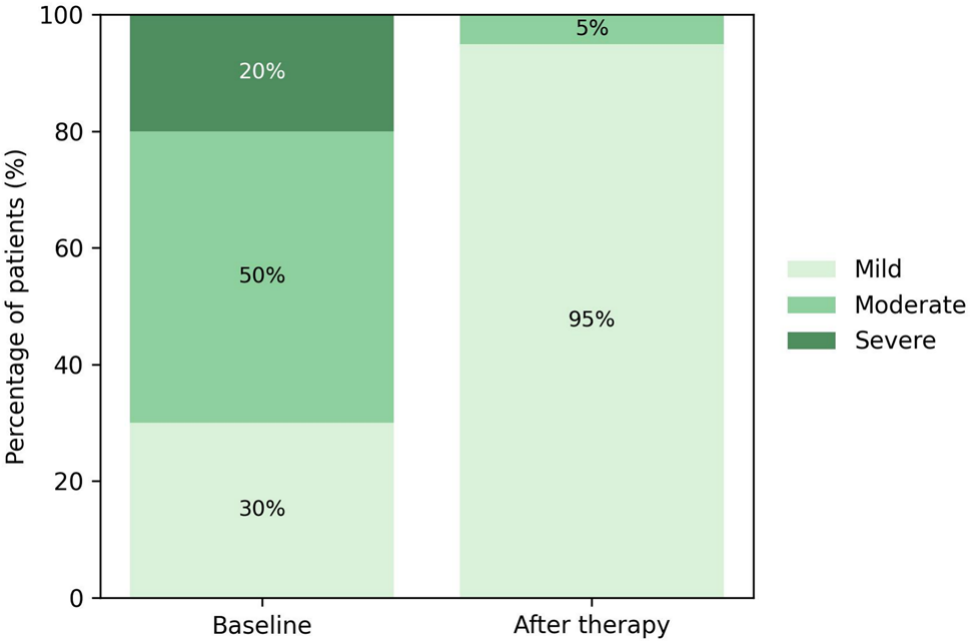
Distribution of DHI severity categories at baseline and after 3 months of structured counselling and multimodal therapy

Categorical analysis demonstrated a marked reduction in moderate and severe dizziness handicap, with most patients shifting into the mild handicap category at follow-up (Fig. 4).

**Fig. 5:**
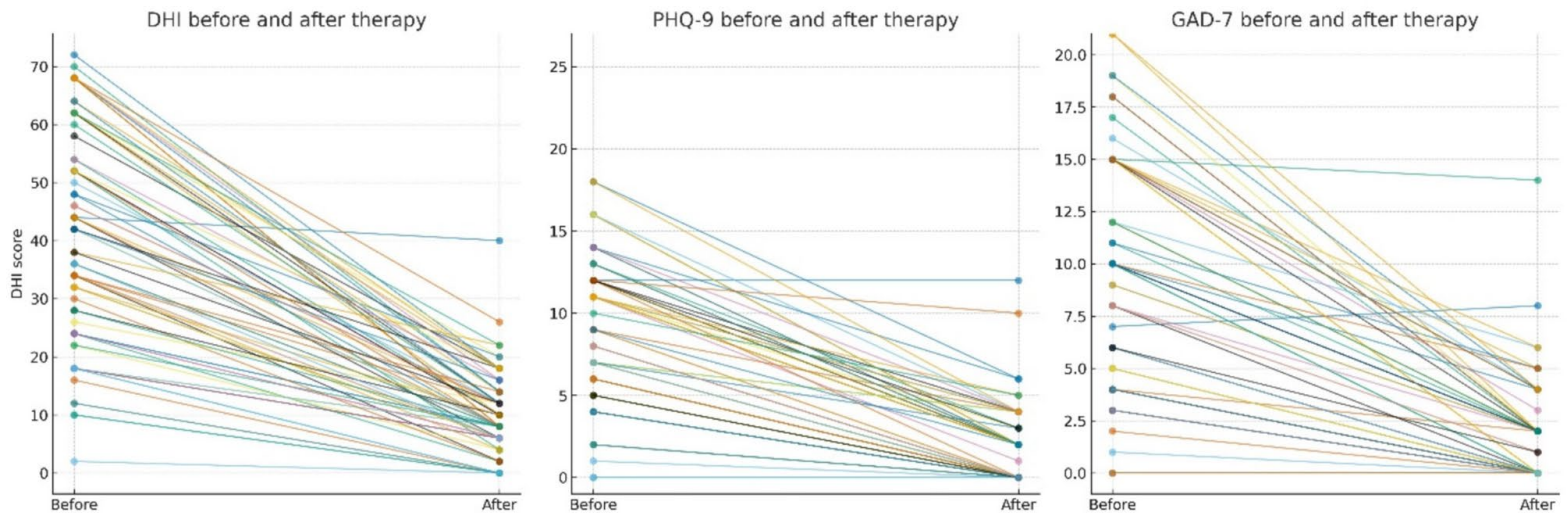
Individual patient trajectories for DHI, PHQ-9, and GAD-7 scores before and after 3 months of structured counselling and multimodal therapy (*n* = 75). Each line represents one patient

Individual patient trajectories demonstrated consistent improvement across dizziness, anxiety, and depressive symptoms, with predominantly downward slopes from baseline to follow-up (Fig. 5). Only one patient showed worsening of anxiety symptoms at follow-up.

### Adherence and safety

All enrolled patients completed the planned 3-month follow-up assessment. Overall adherence to the multimodal treatment framework was high. No serious adverse events were reported. Pharmacotherapy was generally well tolerated; mild, transient adverse effects such as nausea or somnolence occurred in a small number of patients and did not require discontinuation.

## Discussion

This prospective study demonstrates that PPPD is associated with high psychiatric comorbidity, a marked dissociation between subjective disability and objective balance performance, and substantial clinical improvement observed after a structured, neurologist-led multimodal treatment approach. Our findings are consistent with international literature while adding prospective outcome data from low- and middle-income South Asian clinical settings and reinforcing the central, network-based nature of PPPD.

The demographic and clinical profile of our cohort aligns with previous reports, with predominance in middle-aged patients and a higher prevalence in women. Importantly, 42.7% of patients had no identifiable precipitating event, supporting the Bárány Society criteria that do not require a trigger for diagnosis [2]. This has practical implications, as absence of a precipitant often leads to diagnostic uncertainty and unnecessary investigations in routine practice. Our findings indicate that idiopathic onset is common and should not deter clinicians from making a confident diagnosis.

Although the proportion of idiopathic-onset cases may initially appear high, an identifiable precipitating event is not mandatory under Bárány Society criteria. The diagnosis in all cases was based on characteristic positive symptom patterns together with the exclusion of active vestibular or neurological disorders capable of independently explaining symptoms.

Psychiatric comorbidity was present in 88% of patients, most commonly combined anxiety and depression, consistent with prior PPPD cohorts[10, 11, 13]. Importantly, baseline DHI scores did not differ between patients with and without psychiatric comorbidity, and substantial disability persisted even after accounting for mood symptoms. This supports the view that anxiety and depression modulate symptom perception and persistence rather than constituting the primary pathology[1, 9]. The higher PHQ-9 and GAD-7 scores observed in patients with identifiable precipitants may reflect heightened threat appraisal following an acute vestibular or medical event, in keeping with cognitive-behavioral models of PPPD[1, 3, 9].

A key finding of this study was the pronounced subjective–objective dissociation on balance testing. This dissociation between perceived disability and measured postural performance mirrors the phenomenon of postural misperception described in PPPD, in which patients report exaggerated instability despite objectively normal—or even reduced—sway [9, 23]. This reflects altered central estimation of self-motion rather than true balance failure, consistent with contemporary network-based models of PPPD [9, 23]. Despite moderate to severe perceived disability, most posturographic parameters—including sway velocity, ellipse area, and sensory condition-specific stability indices—were within normal limits and showed no correlation with DHI scores. Limit of Stability (LOS) abnormalities were observed in only a small subset of patients with severe disability. These findings reinforce prior observations that conventional balance metrics often fail to capture PPPD severity [1, 5, 7] and highlight the importance of recognizing PPPD as a disorder of maladaptive balance strategies and central sensory processing rather than structural instability. From a clinical perspective, normal posturography should be reassuring rather than contradictory when PPPD is suspected.

The most striking observation was the robust and consistent improvement across all outcome measures following multimodal therapy. Median DHI scores decreased from moderate to minimal disability, and anxiety and depressive symptoms normalized in most patients within three months. These improvements compare favorably with outcomes reported in PPPD cohorts, including CBT-informed vestibular rehabilitation studies and multimodal treatment series [16, 23], while appearing relatively rapid and consistent across patients. Recent systematic reviews and meta-analyses have highlighted substantial heterogeneity in PPPD treatment protocols and relatively limited prospective controlled evidence [14, 15, 23]. The consistency of improvement across group-level, categorical, and individual trajectory analyses in our cohort supports the clinical value of a structured, integrated treatment approach.

Compared with prior studies such as Waterston et al., which reported improvement with CBT-based treatment in a retrospective cohort, our study prospectively evaluated a structured, neurologist-led multimodal treatment approach. Importantly, diagnostic explanation and pre-treatment counselling were standardized and delivered as core components of therapy for all patients. Our findings suggest that treatment response in PPPD may depend not only on the individual modalities used, but also on early diagnostic framing, patient acceptance of the diagnosis, and structured integration of rehabilitation, psychological intervention, and pharmacotherapy when indicated.

A potentially important contributor to these outcomes was the systematic neurologist-delivered diagnostic explanation and pre-treatment counselling provided to all patients. PPPD was framed as a positive, brain-based disorder of maladaptive sensory processing rather than structural damage, with emphasis on reversibility and active participation in rehabilitation. Prior studies have similarly emphasized the importance of patient understanding and acceptance of the diagnosis in facilitating recovery [16]. Our findings further support the concept that the initial neurological consultation itself may function as an early therapeutic intervention in PPPD.

## Strengths and limitations

The strengths of this study include its prospective design, standardized multimodal treatment framework, systematic assessment using validated symptom scales, and integration of objective posturography with clinical and psychiatric evaluation. The study also provides prospective PPPD outcome data from a low- and middle-income South Asian clinical setting, where structured multidisciplinary PPPD management remains relatively underreported.

Several limitations should be acknowledged. First, this was a single-center observational study without a control group, limiting causal inference regarding the relative contribution of individual treatment components. Second, the PPPD diagnosis was made by a single specialist neurologist rather than through independent multidisciplinary adjudication, although all patients fulfilled Bárány Society criteria and underwent detailed neurological and neuro-otological evaluation. Third, outcomes were not separately analyzed in medication-naïve patients versus those already receiving antidepressants or anxiolytic medications before enrolment. Finally, the follow-up duration was limited to three months, and longer-term outcome studies are needed to determine the durability of treatment response.

## Conclusions

This prospective study demonstrates that PPPD is characterized by high psychiatric comorbidity and a marked mismatch between subjective disability and objective balance performance, yet remains highly responsive to treatment. In this Indian cohort, a structured, neurologist-led multimodal approach was associated with substantial improvement in dizziness-related handicap and normalization of anxiety and depressive symptoms within three months.

Structured physician-delivered diagnostic explanation and education may have contributed importantly to these outcomes by framing PPPD as a positive, brain-based functional disorder and preparing patients for vestibular rehabilitation, psychological intervention, and pharmacotherapy when indicated. These findings reinforce that PPPD is not only diagnosable with confidence but also potentially highly treatable when approached with clear communication and an integrated therapeutic framework.

Early recognition and structured pre-treatment education may play an important role in optimizing engagement, adherence, and clinical outcomes in routine neurological practice, including in low- and middle-income settings.

## Data Availability

Raw data files will be provide by the authors upon reasonable request.
